# Enhancing survival in cardiac arrest: the urgent need for optimized extracorporeal cardiopulmonary resuscitation implementation and protocols – editorial

**DOI:** 10.1097/MS9.0000000000002740

**Published:** 2024-11-13

**Authors:** Aiman Waheed, Muhammad H. Gul, Risha Naeem, Hafsa Arshad Azam Raja, Abdul B. Wardak, Umer Khan

**Affiliations:** a Department of Anesthesia, Rawalpindi Medical University and Allied Hospitals, Rawalpindi, Pakistan; b Department of General Medicine, Hayatabad Medical Complex, Peshawar, Pakistan; c Department of Internal Medicine, Ameer-ud-din Medical College, Lahore, Pakistan; d Department of Surgery, Rawalpindi Medical University and Allied Hospitals, Rawalpindi, Pakistan; e Department of Surgery, Razia Bahlol Hospital, Afghanistan, Madina-Township Kabul, Afghanistan; f Peshawar Medical College, Peshawar, Pakistan

**Keywords:** acute medicine, CPR, critical care, ECPR, emergency medicine

## Abstract

Extracorporeal CPR (ECPR) involves venoarterial extracorporeal membrane oxygenation (VA-ECMO) in patients with sudden and unexpected pulse loss due to heart-stopping mechanical activity. ECPR in patients with cardiac arrest has been shown to significantly improve the prognosis. In emergency rooms, interdisciplinary coordination exists among emergency medicine, cardiology, critical care, and perfusion technology. However, some problems must be solved, such as excessive costs, resource allocation, and the need for specialized equipment. Resuscitation success and patient outcomes can be enhanced by combining ECPR and emergency care.

Extracorporeal CPR (ECPR) represents a groundbreaking advance in resuscitation technology that utilizes venoarterial extracorporeal membrane oxygenation (VA-ECMO) to offer a lifeline for patients who experience sudden and severe cardiac arrest^1^. By bolstering cerebral blood flow and supporting neurological recovery, ECPR has transformed the prognosis for those who would otherwise face dismal survival chances^2^. Compared to conventional treatments such as manual CPR, defibrillation, and advanced cardiovascular life support (ACLS), ECPR offers a significant improvement in survival rates, particularly at 30 days, for patients experiencing refractory cardiac arrest^3^. Coupled with targeted temperature management (TTM), it significantly improves outcomes in out-of-hospital cardiac arrest (OHCA) patients by turning the tide in the fight against one of the most critical emergencies in medicine^1^. Predicting outcomes for refractory cardiac arrest in prehospital settings remains challenging despite the development of prediction models for post-OHCA ICU patients^3^. ECPR is generally considered for patients aged <75 years who have experienced cardiac arrest, present with a shockable rhythm, and have a brief duration of cardiac arrest. These criteria are commonly used to determine the eligibility^4^. Refractory cardiac arrest (RCA) due to reversible factors was the primary criterion for inclusion^4,5^. Major comorbidities, advanced cancer, active bleeding, and severe neurological damage were commonly used exclusion criteria^5^. The steps involved in ECPR are illustrated in Figure 1.

**Figure 1:**
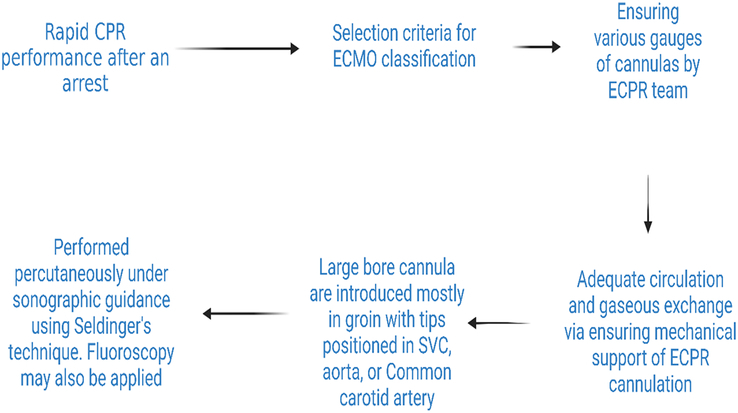
Steps involved in ECPR.

For extracorporeal CPR (ECPR), femoral vessel cannulation is commonly used because of its accessibility during ongoing cardiopulmonary resuscitation (CPR). Alternative cannulation routes, such as jugular-femoral, femoral-subclavian, and jugular-subclavian, may also be employed depending on the patient’s condition and available resources. Implementing ECPR outside the operating room requires specialized equipment and a trained team. According to the American Heart Association (AHA) guidelines, evaluating neurological outcomes in patients undergoing ECPR involves a multifaceted approach that includes physical examinations, electrophysiological tests, imaging studies, and laboratory markers^6^. This comprehensive evaluation includes assessing signs such as pupil size and brainstem reflexes, performing imaging techniques such as brain CT scans and MRI, conducting electrophysiological studies such as electroencephalogram (EEG) and bispectral index monitor (BIS), and analyzing blood markers such as arterial pH and serum lactate levels. These tools are crucial for predicting patient outcomes and guiding decisions during ECPR^6^. While central cannulation can be achieved through sternotomy by a cardiothoracic surgeon, peripheral access is often performed percutaneously in the ICU or during cardiac catheterization^7^.

Extracorporeal membrane oxygenation (ECMO) can be performed in two configurations: venovenous ECMO (VV-ECMO) and venoarterial ECMO (VA-ECMO). VV-ECMO provides respiratory support by oxygenating blood from the venous system and returning it to the venous system, making it effective for patients with severe respiratory failure unresponsive to conventional ventilation. In contrast, VA-ECMO offers both respiratory and cardiac support, making it suitable for patients with cardiac arrest, severe cardiogenic shock, or difficulty weaning off cardiopulmonary bypass postcardiac surgery, with survival rates ranging from 20 to 30%^7^. VA-ECMO withdraws blood from the right atrium or femoral vein and reinfuses it into the arterial system, typically through the femoral artery or directly into the aorta after oxygenation. While VA-ECMO effectively supports both cardiac and respiratory functions, it carries a higher risk of complications such as neurological damage, stroke, and blood loss compared to VV-ECMO. However, patients receiving VA-ECMO have shown significantly lower in-hospital mortality, reduced neurological complications, and shorter durations of ECMO and mechanical ventilation^6,7^. Optimizing ECPR protocols is essential to improving survival and minimizing adverse outcomes in cardiac arrest scenarios.

To provide respiratory support but not cardiac support, VV ECMO removes blood from the venous system, oxygenates it, and then puts it back in. VA ECMO, on the other hand, provides cardiac and respiratory support by drawing blood from the venous system and reinfusing it into the arterial system^8^. Thorough cardiopulmonary assistance is essential when standard techniques are insufficient to treat cardiac arrests. Maintaining a core body temperature between 33°C and 35°C for 24–48 h is advised, with oxygenation and ventilation strategies adjusted based on individual patient needs^9^. Targeted temperature management (TTM) is a critical component, as hyperthermia is frequently observed and associated with poorer neurological outcomes if not managed promptly^6,9^. Improvement in patient outcomes after ECPR depends on effective patient management^6^. Although the rates of successful weaning, bleeding, bloodstream infections, and pump failure are similar for both VV and VA ECMO, optimizing ECPR protocols could improve patient survival and recovery^8^. Despite the encouraging findings of numerous studies, more research is still needed to fully understand the pathophysiology and serious side effects of ECPR, such as ischemia, infection, and blood loss^1^. Figure 2 shows the workflow from cardiac arrest to ECMO initiation and highlights the crucial steps in optimizing ECPR implementation.

**Figure 2:**
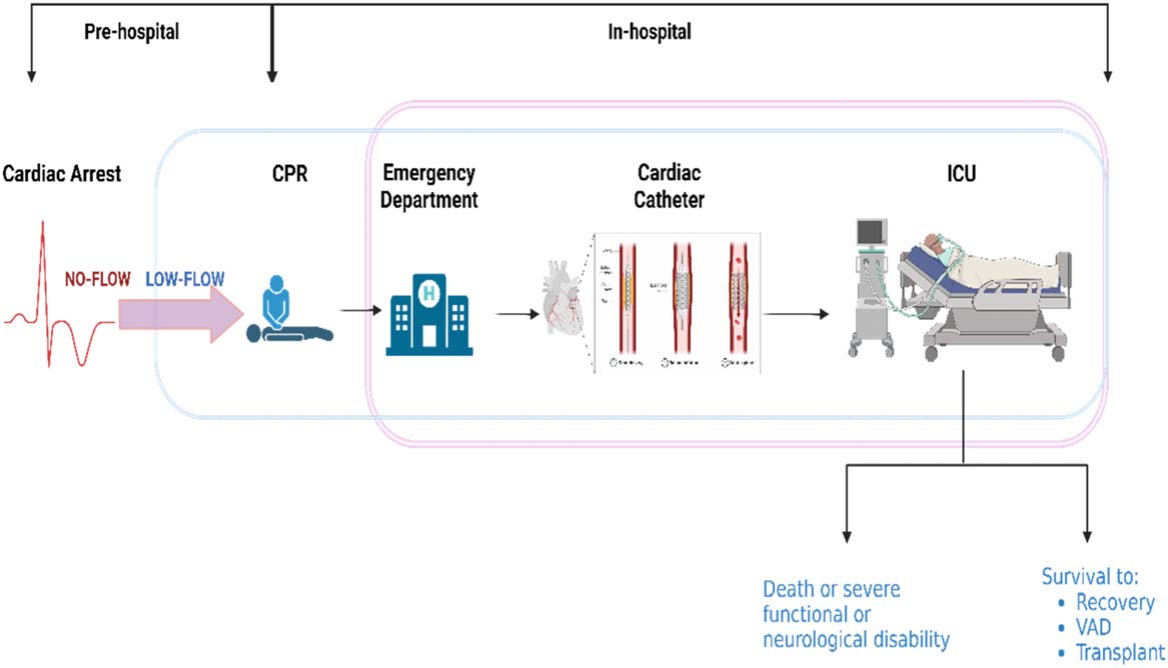
ECPR workflow.

Patients with cardiac arrest undergoing ECPR have shown higher endurance rates than those undergoing conventional CPR. A meta-analysis of observational revisions revealed that ECPR substantially improved the likelihood of endurance until hospital discharge in patients with in-hospital cardiac arrest^2^. This is due to ECPR’s ability to maintain end-organ perfusion while addressing the underlying cause of cardiac arrest, potentially lowering the mortality associated with prolonged low-flow conditions^10^. However, there is a risk of severe neurological impairment in survivors of cardiac arrest, despite some studies suggesting that ECPR can improve neurological outcomes due to better preservation of cerebral perfusion during cardiac arrest^11^. The length of low-flow states before ECPR begins and the patient’s pre-existing medical issues frequently impact the variety of outcomes^12^. While a small percentage of ECPR survivors experienced satisfactory neurological status, a significant proportion had moderate to severe deficits, which raises ethical and clinical questions regarding the widespread use^13^.

One of the biggest obstacles in ECPR is the choice of suitable candidates. Not every patient with a cardiac arrest is a good fit for ECPR. Younger patients with witnessed cardiac arrest, short no-flow times, and cases of reversible cardiac arrest—where the underlying cause can be identified and treated, such as hypoxia, hypovolemia, electrolyte imbalances, or tension pneumothorax—are generally the best candidates^3^. To optimize the benefits of ECPR, the selection procedure needs to be quick and precise; however, this is frequently made more difficult by the erratic nature of emergency care settings and the variety of patient presentations. Timely initiation of ECPR is critical for its success. As extended durations of low-flow states are linked to worse outcomes, delaying the onset of ECPR can significantly reduce its efficacy^14^. Reducing the time from cardiac arrest to ECPR initiation is vital and depends on having well-equipped, trained staff and advanced equipment. Multidisciplinary coordination in ECPR involves collaboration among various healthcare professionals, including emergency physicians, cardiologists, cardiothoracic surgeons, intensivists, anesthesiologists, perfusionists, and nurses. Each team member plays a crucial role in the process, from initial resuscitation to ECMO management and patient recovery. For example, the emergency physician leads the resuscitation efforts and assesses ECPR eligibility, while the surgeon performs cannulation, and perfusionists manage the ECMO machine. This coordinated teamwork, supported by continuous training and simulation, enhances patient care and improves survival, and recovery outcomes. Future studies should focus on improving patient selection, timing, post-ECPR care, cost-effectiveness, and standardizing protocols to enhance patient outcomes^15^.

In conclusion, it should be noted that performing extracorporeal cardiopulmonary resuscitation (ECPR) on patients who are having cardiac arrest has been demonstrated to significantly improve their prognosis. When a patient does not respond to traditional CPR, it is a valuable alternative that shows possible improvements in neurological rehabilitation and survival. Timely and efficient treatment implementation necessitates a diverse team, specialized training, and strict adherence to established guidelines. However, some problems must be solved, such as excessive costs, resource allocation, and the need for specialized equipment. Resuscitation success and patient outcomes can be enhanced by combining ECPR and emergency care.

## Ethical approval

Ethics approval was not required for this editorial.

## Consent

Informed consent was not required for this editorial.

## Source of funding

The authors received no funding for the study.

## Author contribution

M.H.G., U.K., and A.W.: conceptualization; R.N. and H.A.A.R.: literature and drafting of the manuscript; A.B.W.: editing and supervision. All authors have read and agreed to the final version of the manuscript.

## Conflicts of interest disclosure

The authors declare no potential conflicts of interest concerning the research, authorship, or publication of this article.

## Research registration unique identifying number (UIN)

Not applicable.

## Guarantor

Not applicable.

## Data availability statement

Not applicable.

## Provenance and peer review

Not applicable.

## References

[1] Inoue A Hifumi T Sakamoto T . Extracorporeal cardiopulmonary resuscitation for out‐of‐hospital cardiac arrest in adult patients. J Am Heart Assoc 2020;9:e015291. 32204668 10.1161/JAHA.119.015291 PMC7428656

[2] Sakamoto T Morimura N Nagao K . Extracorporeal cardiopulmonary resuscitation versus conventional cardiopulmonary resuscitation in adults with out-of-hospital cardiac arrest: a prospective observational study. Resuscitation 2014;85:762–768. 24530251 10.1016/j.resuscitation.2014.01.031

[3] Debaty G Babaz V Durand M . Prognostic factors for extracorporeal cardiopulmonary resuscitation recipients following out-of-hospital refractory cardiac arrest. A systematic review and meta-analysis. Resuscitation 2017;112:1–10. 28007504 10.1016/j.resuscitation.2016.12.011

[4] Otani T Sawano H Hayashi Y . Optimal extracorporeal cardiopulmonary resuscitation inclusion criteria for favorable neurological outcomes: a single‐center retrospective analysis. Acute Med Surg 2019;7:e447. 31988761 10.1002/ams2.447 PMC6971448

[5] Koen’TJ Nathanaël T Philippe D . A systematic review of current ECPR protocols. A step towards standardisation. Resusc Plus 2020;3:100018. 34223301 10.1016/j.resplu.2020.100018 PMC8244348

[6] Springer Nature Link . ECPR—extracorporeal cardiopulmonary resuscitation | Indian Journal of Thoracic and Cardiovascular Surgery. Accessed 29 June 2024. https://link.springer.com/article/10.1007/s12055-020-01072-2#Abs1

[7] NIH . Extracorporeal Membrane Oxygenation in Adults - StatPearls - NCBI Bookshelf. Accessed 29 June 2024. https://www.ncbi.nlm.nih.gov/books/NBK576426/

[8] NIH . Venovenous vs. Venoarterial Extracorporeal Membrane Oxygenation in Infection-Associated Severe Pediatric Acute Respiratory Distress Syndrome: A Prospective Multicenter Cohort Study - PMC. Accessed 29 June 2024. https://www.ncbi.nlm.nih.gov/pmc/articles/PMC8982932/

[9] Olson T Anders M Burgman C . Extracorporeal cardiopulmonary resuscitation in adults and children: A review of literature, published guidelines and pediatric single-center program building experience. Front Med 2022;9:935424. 10.3389/fmed.2022.935424 PMC9720280 36479094

[10] Chen YS Lin JW Yu HY . Cardiopulmonary resuscitation with assisted extracorporeal life-support versus conventional cardiopulmonary resuscitation in adults with in-hospital cardiac arrest: an observational study and propensity analysis. Lancet Lond Engl 2008;372:554–561. 10.1016/S0140-6736(08)60958-7 18603291

[11] Fagnoul D Combes A De Backer D . Extracorporeal cardiopulmonary resuscitation. Curr Opin Crit Care 2014;20:259–265. 24785674 10.1097/MCC.0000000000000098

[12] Thiagarajan RR Laussen PC Rycus PT . Extracorporeal membrane oxygenation to aid cardiopulmonary resuscitation in infants and children. Circulation 2007;116:1693–1700. 17893278 10.1161/CIRCULATIONAHA.106.680678

[13] Pozzi M Koffel C Armoiry X . Extracorporeal life support for refractory out-of-hospital cardiac arrest: Should we still fight for? A single-centre, 5-year experience. Int J Cardiol 2016;204:70–76. 26655543 10.1016/j.ijcard.2015.11.165

[14] Maekawa K Tanno K Hase M . Extracorporeal cardiopulmonary resuscitation for patients with out-of-hospital cardiac arrest of cardiac origin: a propensity-matched study and predictor analysis. Crit Care Med 2013;41:1186–1196. 23388518 10.1097/CCM.0b013e31827ca4c8

[15] Makdisi G Wang I-wen . Extra Corporeal Membrane Oxygenation (ECMO) review of a lifesaving technology. J Thorac Dis 2015;7:E166–E176. 26380745 10.3978/j.issn.2072-1439.2015.07.17 PMC4522501

